# Endocytosis of Peptidase Inhibitor SerpinE2 promotes Myocardial Fibrosis through activating ERK1/2 and β-catenin Signaling Pathways

**DOI:** 10.7150/ijbs.67726

**Published:** 2022-10-17

**Authors:** Chao Li, Li-fang Lv, Mu-ge Qi-Li, Rui Yang, Yu-jing Wang, Shuang-shuang Chen, Ming-xiu Zhang, Tian-yu Li, Tong Yu, Yu-hong Zhou, Hai-hai Liang, Hong-li Shan, Xue-lian Li

**Affiliations:** 1 Department of Pharmacology (State-Province Key Laboratories of Biomedicine-Pharmaceutics of China, Key Laboratory of Cardiovascular Research, Ministry of Education), College of Pharmacy, Harbin Medical University, Harbin, Heilongjiang, P. R. China.; 2 The Centre of Functional Experiment Teaching, School of Basic Medicine, Harbin Medical University, Harbin, Heilongjiang 150081, P. R. China.; 3 Department of Pharmacy, Sunshine Union Hospital, Shandong Weifang, China.; 4 Research Unit of Noninfectious Chronic Diseases in Frigid Zone (2019RU070), Chinese Academy of Medical Sciences, Harbin, 150081, China.; 5 Shanghai Frontiers Science Research Center for Druggability of Cardiovascular Noncoding RNA, Institute for Frontier Medical Technology, Shanghai University of Engineering Science, Shanghai, 201620, China.

**Keywords:** Cardiac Fibrosis, SerpinE2, Endocytosis, ERK1/2, β-catenin signaling

## Abstract

Cardiac fibrosis is one of the common pathological processes in many cardiovascular diseases characterized by excessive extracellular matrix deposition. SerpinE2 is a kind of protein that inhibits peptidase in extracellular matrix and up-regulated tremendously in mouse model of cardiac fibrosis induced by pressure-overloaded via transverse aortic constriction (TAC) surgery. However, its effect on cardiac fibroblasts (CFs), collagen secretion and the underlying mechanism remains unclear. In this study, DyLight® 488 green fluorescent dye or His-tagged proteins were used to label the exogenous serpinE2 protein. It was showed that extracellular serpinE2 translocated into CFs by low-density lipoprotein receptor-related protein 1 (LRP1) and urokinase plasminogen activator receptor (uPAR) of cell membrane through endocytosis. Knockdown of LRP1 or uPAR reduced the level of serpinE2 in CFs and down-regulated the collagen expression. Inhibition of the endocytosis of serpinE2 could inhibit ERK1/2 and β-catenin signaling pathways and subsequently attenuated collagen secretion. Knockdown of serpinE2 attenuates cardiac fibrosis in TAC mouse. We conclude that serpinE2 could be translocated into cardiac fibroblasts due to endocytosis through directly interact with the membrane protein LRP1 and uPAR, and this process activated the ERK1/2, β-catenin signaling pathways, consequently promoting collagen production.

## Introduction

Myocardial fibrosis is a malignant pathophysiological process of the heart characterized by cardiac stiffness, which ultimately affects cardiac systolic and diastolic function 1. Cardiac fibrosis is defined as the excess deposition of extracellular matrix (ECM), particularly collagen, around myocytes and vascular cells. Several types of cells exist in the heart, such as myocytes, fibroblasts, immune cells, and endothelial cells 2, 3, among which fibroblasts have long been recognized as the major cell population. As the activation of cardiac fibroblasts, collagen is excessively synthesized, secreted into the extracellular space, and eventually led to abundant collagen deposition as the extracellular matrix.

SerpinE2 is member 2 of the serpin peptidase inhibitor clade E, also known as glia-derived nexin (GDN) and protease nexin 1 (PN-1). SerpinE2 is a protease inhibitor that is secreted by fibroblasts and several other cells 4. It is mainly located in the extracellular space, extracellular vesicles, extracellular matrix, and plasma membrane. SerpinE2 in blood cells (monocytes and platelets) or vessels (endothelial and muscle cells) has an impact on thrombus formation, thrombolysis, and vascular hyperpermeability 5. In our previous study, serpinE2 was up-regulated in mouse fibrosis model both *in vivo* and *in vitro* observations, moreover, knockdown of serpinE2 could attenuate cardiac fibrosis in the mouse model of TAC. Furthermore, it was found that serpinE2 is expressed mainly in myocardial fibroblasts induced by Ang II or TGF-β, and exogenous serpinE2 increased the content of collagen 6. However, the molecular mechanism insight into the function of serpinE2 on cardiac fibrosis remains elusive.

It has reported that serpinE2 can be translocated into the cells via uPAR or LRP pathway in prostate tumor cells 7. SerpinE2 combines with uPA to form a complex that can be internalized by LRP pathway during cerebellar development 8. SerpinE2 in the blood circulation can be quickly eliminated by the LRP receptor on the cell surface after entering into the cells 9. It has also been reported that, serpinE1, an analog of Serpine2, can activate β-catenin and ERK/MAPK pathways to promote cell migration through LRP1 endocytosis 10. The uPA-serpinE1 complex also activates the PKA signaling pathway through uPAR/LRP and increases lung permeability 11. Does serpinE2 undergo endocytosis and activate the signaling pathways similar to serpinE1?

Based on previous studies, the purpose of this study is to provide new evidence to demonstrate the functional role of exogenous serpinE2 on cardiac fibroblasts and the molecular mechanism of serpinE2 in the regulation of cardiac fibrosis.

## Materials and Methods

### Cardiac fibroblasts

Cardiac fibroblasts were isolated from 1-3-day old neonatal mice as described previously 12. Briefly, cardiac fibroblasts were separated by the removal of cardiac myocytes through selective adhesion of non-myocytes at a 1.5 h pre-plating interval. Then cardiac fibroblasts were maintained in DMEM (Biological Industries, BI, USA) with 10% fetal bovine serum (Biological Industries, BI, USA) in 5% CO_2_ in 37 ºC. Transfections of LRP-1 or uPAR siRNA (Guangzhou Ribo Bio Co. Ltd., Guangzhou, China) were performed according to the manufacturer's protocol. SerpinE2 antibody (SerpinE2 antibody, rabbit polyclonal, ab75348, Abcam Cambridge, MA, USA) and heparin (low dose 125 U, high dose 250 U, Hangzhou Jiuyuan Gene Engineering Co., Ltd., Hangzhou, China) were used to combine with exogenous serpinE2; Nystatin (10 ng/mL, Sigma-Aldrich, St. Louis, MO, USA) and β-cyclodextrin (0.5 mmol/L, Shanghai Aladdin Biochemical Technology Co., Ltd., Shanghai, China) were used to inhibit the endocytosis of cardiac fibroblasts *in vitro* preparation.

### Western blotting

The total protein was extracted from cardiac fibroblasts or cardiac tissues, and then fractionated by 10% SDS-PAGE and transferred to NC membranes. After that, membranes were incubated with primary antibodies overnight at 4 °C before they were washed and incubated with secondary antibodies (Alexa Fluor800 or 680, Abcam). The primary antibodies such as anti-β-catenin, anti-p- ERK1/2 (anti-p- ERK1/244/42), anti-T- ERK1/2 (anti-T-ERK44/42) and anti-SerpinE2 were obtained from Cell Signaling (Danvers, MA, USA). Anti-Collagen I was obtained from Santa Cruz Biotechnology (Santa Cruz, CA, USA). Anti-GAPDH was obtained from OriGene (Rockville, MD, USA). GAPDH was used as an internal control. Bands were quantified by Odyssey v1.2.

### Real-time RT-PCR

Total RNA was extracted using TRIZOL reagent (Invitrogen, Carlsbad, CA, USA) from cardiac tissues and cardiac fibroblasts according to the manufacturer's instructions. The RNA levels for Collagen I, Collagen III, α-SMA and β-catenin were quantified by fast real-time PCR system (ABI 7500, Applied Biosystems, Carlsbad, CA, USA). GAPDH was set as an internal control. The 2^-ΔΔCt^ method was applied for the data analysis and the data were normalized and converted into relative mRNA expression. The primers used in this study were shown in Table 1.

### Masson staining

Mice hearts were first fixed in 4% paraformaldehyde solution for 24h at room temperature. Then hearts were embedded in paraffin and sectioned into 5 μM for further study. Masson staining was performed using Masson's Trichrome Stain Kit (Solarbio, China). Myocardial tissues were stained in red while Collagen stained in blue. All quantitative evaluations were carried out using ImagePro Plus software (version 6.0, Media Cybernetics, Bethesda, MD).

### Immunofluorescence

Intracellular p-ERK and β-catenin expression were observed via immunofluorescence. Cardiac fibroblasts were first fixed by 4% paraformaldehyde solution for 20 min and then permeabilizated by 0.1% Triton-X100 solution. CFs were blocked by goat serum for 1 h before incubated with primary antibody against p-ERK or β-catenin overnight at 4 °C. After washed with PBS, incubated cells were then incubated with secondary antibodies Alexa Fluor® 594 (1:1000, Invitrogen, Waltham, MA, USA).

To examine the endocytosis of serpinE2 into cardiac fibroblasts, exogenous serpinE2 (recombinant mouse serpinE2 protein, ab92712) containing primary amines was covalently labeled with DyLight® 488 Fast Conjugation Kit (ab201799, Abcam) according to the manufacturer's instructions. DyLight® 488 has strong absorption at 493 nm, high fluorescence at 518 nm, and high quantum yield. It can covalently label proteins, peptides, and other biomolecules containing primary amines. SerpinE2 is rich in lysine and arginine, containing a large amount of primary amine. Exogenous serpinE2 and DyLight®488 modifier reagent were mixed to form a conjugated complex. Nuclei were counterstained with DAPI (1:50, Roche Diagnostics, Basel, Switzerland) for 5 min at room temperature. Immunofluorescence was visualized under a fluorescence microscope (Nikon 80i, Nikon Corporation, Tokyo, Japan).

We also used serpinE2-His Tag (SE2-H5225, Acrobiosystems, Newark, USA) to detect the endocytosis of serpinE2. This protein carries a His tag at the C-terminus. The His-tag antibody was used to demonstrate the protein level. The fluorescence second antibody was used. Nuclei were counterstained with DAPI (1:50, Roche Diagnostics, Basel, Switzerland) for 5 min at room temperature. Immunofluorescence was visualized under a fluorescence microscope (Nikon 80i, Nikon Corporation, Tokyo, Japan).

### Co‑immunoprecipitation (Co-IP)

Cells were lysed by IP lysis buffer (Beyotime, Shanghai, China). The protein complex wascaptured by the Anti-serpinE2 antibody (Cell Signaling Technology, MA, USA) and Anti-rabbit IgG antibody (Cell Signaling Technology, MA, USA). Protein A/G PLUS-Agarose (Santa Cruz Biotechnology, Dallas, USA) was used to precipitate the protein complex that combined with the antibody and washed with PBS. Then, the protein sample was detected by immunoblotting. The uPAR was explored with antibody (Proteintech, Wuhan, China) and LRP1 was explored with antibody (Proteintech, Wuhan, China). IgG was used as a negative control.

### Quantification of collagen

Total collagen content was measured using a Sircol™ soluble collagen assay kit (Biocolor Ltd., Carrickfergus, U.K.) according to the manufacturer's protocol. The Sircol™ assay is a dye-binding method designed for the analysis of acid and pepsin-soluble collagen. Briefly, dye solutions were collected from each group and the collagen content was calculated using a microplate reader (Sunrise™, Tecan Trading AG, Männedorf, Switzerland).

### Enzyme-linked immunosorbent assay (ELISA)

The levels of β-catenin and ERK1 were measured using the Mouse CTNNb1 (Catenin, Beta 1) ELISA Kits and Mouse ERK1 (Extracellular Signal Regulated Kinase 1) ELISA Kits (Elabscience Biotechnology, Wuhan, China) according to the manufacturer's protocol. The levels of serpinE2 in plasma, culture medium (supernatant of fibroblasts) and tissue homogenates were measured by ELISA kits (Elabscience Biotechnology, Wuhan, China) according to the manufacturer's instructions.

### Pressure overload-induced cardiac fibrosis mouse model

8-week-old male C57BL/6 mice were purchased from the Experimental Animal Center of the Second Affiliated Hospital of Harbin Medical University (Harbin, China). Mice were weighed and anaesthetized with 2% avertin in solution, then subjected to pressure overload by surgical transverse aortic constriction (TAC) to induce cardiac fibrosis as described previously 13. Four weeks after TAC, mice hearts were harvested and prepared for subsequent study. All experimental procedures performed in studies involving animal participants were in accordance with the Institutional Animal Care and Use Committee of Harbin Medical University, China and the Guide for the Care and Use of Laboratory Animals, published by the US National Institutes of Health (NIH Publication No. 85-23, revised 1996).

### Knockdown of serpinE2 *in vivo*

Mice were randomly divided into several groups and injected with 1 × 10^7^ TU of either Lentivirus-SerpinE2-RNAi (GeneChem, Shanghai, China) or negative control lentivirus via the tail vein. TAC was performed 7 days after injection.

### Statistical analysis

Averaged data are expressed as mean ± SEM. Paired Student's t tests were applied for the difference between two groups. Statistical comparisons among multiple groups were performed using one-way analysis of variance (ANOVA), and Bonferroni's multiple comparisons test or Tukey's multiple comparisons test were performed according to different grouped experiments. *P* < 0.05 was considered to indicate a statistically significant difference. Statistical values were calculated and illustrated using GraphPad Prism 8.2.1. software.

## Results

### Effects of serpinE2 on collagen production in CFs

**Figure 1:**
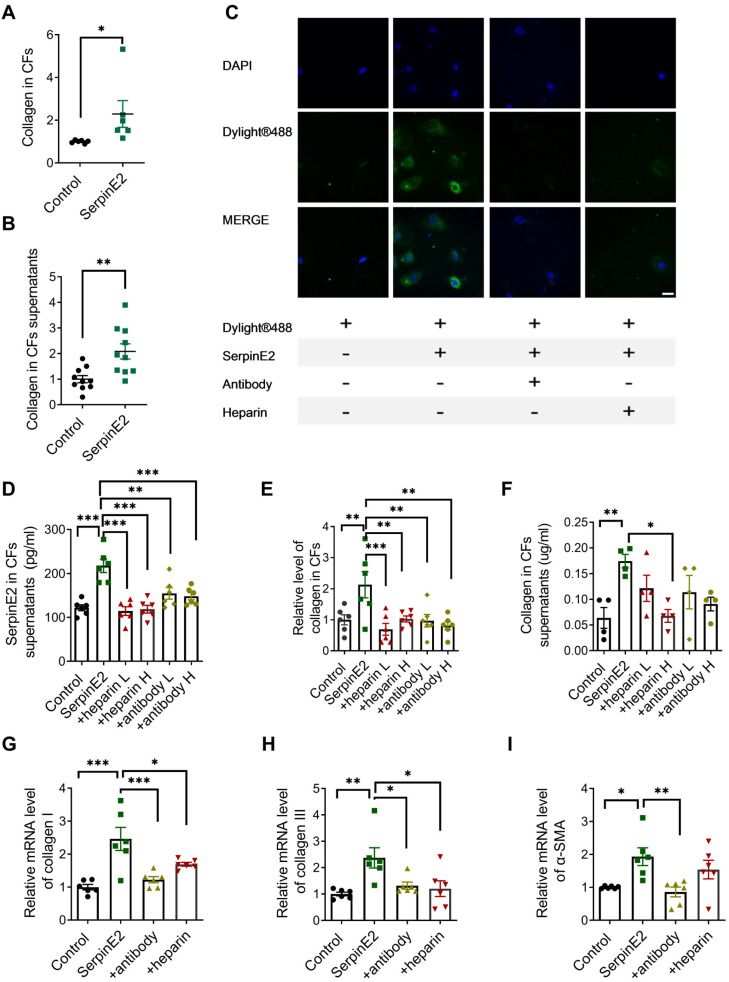
** SerpinE2 promotes collagen expression in cardiac fibroblasts. A-B.** Quantification of collagen content in cardiac fibroblasts and its supernatants after exogenous serpinE2 administration (*n* = 6-10, 20 ng/mL serpinE2 for 48 h); **C.** Immunofluorescence assay showed the distribution of exogenous serpinE2 in cardiac fibroblasts (*n* = 4, Scale bars = 20 µm). **D.** Relative level of serpinE2 in supernatants of fibroblast treated with heparin and serpinE2-antibody (serpinE2 to anti-serpinE2 at a ratio of 1:1 low dose or 1:2 high dose; heparin low dose 125 U, high dose 250 U; *n* = 6); **E-F.** Relative level of collagen in cardiac fibroblasts and supernatants of fibroblasts (*n* = 4 - 6); **G-I.** mRNA level of collagen I, collagen III and α-SMA after treated with serpinE2-antibody or heparin (*n* = 6). Data are presented as mean ± SEM. *p < 0.05, **p < 0.01, ***p < 0.001, determined by student's paired t test (A-B), one-way ANOVA, Bonferroni's multiple comparisons test (D-I).

It was demonstrated that serpinE2 is markedly elevated in TAC-induced fibrosis model in our previous study 6. However, the effects and mechanisms of serpinE2 on collagen production were not clear. To clarify this issue, exogenous serpinE2 protein was applied on cardiac fibroblasts for 48 h to observe its effects on collagen production. After the administration of 20 ng/mL serpinE2, the collagen content either in the cardiac fibroblasts or the supernatants was increased significantly (Figure 1A-B).

DyLight® 488 green fluorescent dye was used to label the exogenous serpinE2 protein. Exogenously administered serpinE2 protein could be internalized into cardiac fibroblasts as detected by increased fluorescence density. In stark contrast, the level of serpinE2 in CFs was significantly down-regulated after administration of serpinE2 antibody or heparin sodium (Figure 1C).

After exogenous addition of serpinE2, the level of serpinE2 was up-regulated in cardiac fibroblasts. In contrast, addition of anti-serpinE2 antibody or heparin sodium to form complexes could suppress the up-regulation of serpinE2 in cardiac fibroblast (Figure 1D), suggesting that serpinE2 is internalized into cardiac fibroblast. Collagen was increased in cardiac fibroblasts and its supernatant after administration of exogenous serpinE2. However, the up-regulation of collagen was suppressed by extracellular serpinE2-antibody complex or serpinE2-heparin complex (Figure 1E-F). These results strongly implied that the exogenous serpinE2 as the part of a complex which is closely associated with the amount of collagen secreting could be reduced in cardiac fibroblasts. Furthermore, RT-PCR results showed that the mRNA levels for collagen I, III and α-SMA were increased after exogenous addition of serpinE2. Collagen I, III and α-SMA mRNA were all decreased after addition of serpinE2-antibody; collagen I, III were also decreased after addition of heparin when compared with serpinE2 group (Figure 1G-I).

### SerpinE2 can be endocytosed into cardiac fibroblasts

**Figure 2:**
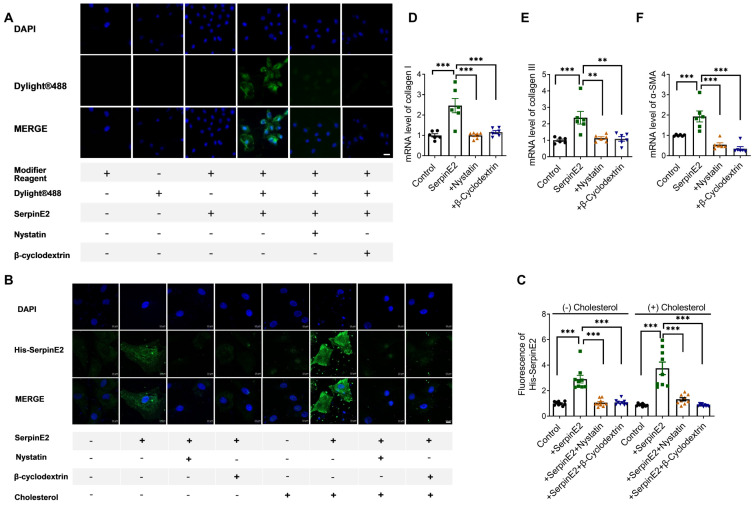
** SerpinE2 can be internalized into cardiac fibroblasts. A.** Immunofluorescence assay demonstrated that nystatin and β-cyclodextrin inhibited the endocytosis of SerpinE2 into cardiac fibroblasts (*n* = 4, Scale bars = 20 µm). **B-C.** SerpinE2 was marked by His tag at the C-terminus. Immunofluorescence assay was used to detect the level of His-SerpinE2 and the statistical graph. (*n* = 9, Scale bars = 10 µm). **D-F.** mRNA level of collagen I, collagen III and α-SMA. (*n* = 6); Data are presented as mean ± SEM. **p < 0.01, ***p < 0.001, determined one-way ANOVA, Bonferroni's multiple comparisons test (C-F).

To further explore if serpinE2 could be internalized into cardiac fibroblasts through endocytosis. SerpinE2 labeled DyLight® 488 was incubated with nystatin or β-cyclodextrin which could inhibit endocytosis (nystatin and β-cyclodextrin has been shown to inhibit the lipid-raft dependent endocytosis by depleting cholesterol on the cell membrane). As detected by fluorescence microscopy, exogenously administered serpinE2 could be internalized into cardiac fibroblasts. As expected, the level of serpinE2 in cardiac fibroblasts (Figure 2A) was significantly reduced by administration of endocytosis inhibitors nystatin and β-cyclodextrin. In order to further confirm if serpinE2 internalized through lipid-raft-dependent endocytosis pathways, serpinE2 was marked by a His tag at the C-terminus, then immunofluorescence was detected under normal condition or cholesterol treatment condition (lipid-raft-dependent endocytosis is sensitive to cholesterol depletion). As shown in Figure 2B, C, administered serpinE2 was internalized into cardiac fibroblasts, nystatin and β-cyclodextrin inhibit the internalization of serpinE2 under both normal condition and cholesterol condition. RT-PCR results also showed that inhibition the endocytosis of serpinE2 down-regulated the mRNA expression level of serpinE2 mediated collagen I, III and α-SMA up-regulation (Figure 2D-F). These observations suggested that nystatin and β-cyclodextrin is very likely to inhibit the translocation of exogenous serpinE2 into cardiac fibroblasts through lipid-raft-dependent endocytosis pathways and lead the down-regulation of collagen expression in cardiac fibroblasts.

### Cell membrane receptors uPAR and LRP1 participate in the endocytosis of serpinE2 into cardiac fibroblasts

**Figure 3:**
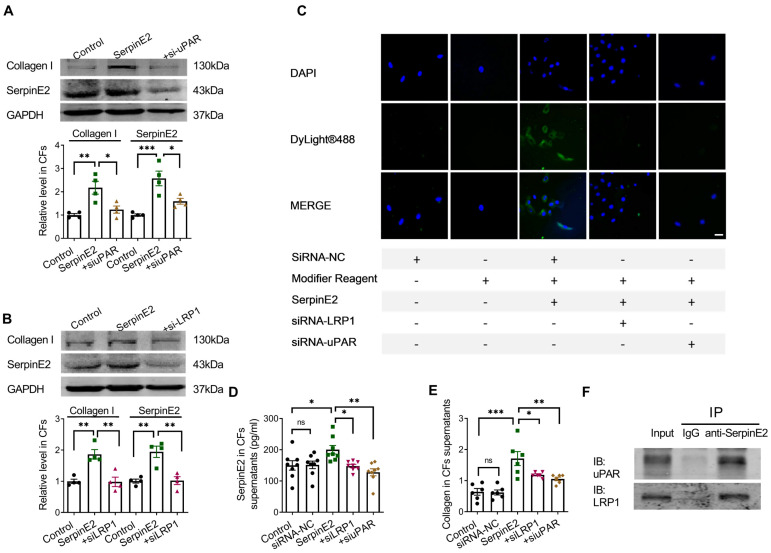
** The effect of LRP1 and uPAR on endocytosis of serpinE2 in cardiac fibroblasts. A.** Protein expression level of collagen I and serpinE2 in cardiac fibroblasts after silencing uPAR (*n* = 4); **B.** Protein expression level of collagen I and serpinE2 in cardiac fibroblasts after silencing LRP1 (*n* = 4); **C.** Immunofluorescence assay demonstrated that knockdown of LRP1 and uPAR reduced endocytosis of serpinE2 into cardiac fibroblasts (*n* = 4, Scale bars = 20 µm); **D.** The level of serpinE2 in supernatant of fibroblast after knockdown of LRP1 and uPAR (*n* = 8); **E.** The content of collagen in supernatant of fibroblast after knockdown of LRP1 or uPAR (*n* = 6); **F.** It was showed the direct interaction between serpinE2 and uPAR, serpinE2 and LRP1 in cardiac fibroblasts by Co-IP. The panel shows the presence of uPAR and LRP1 in the sample pulled down by anti-serpinE2. Data are presented as mean ± SEM. *p < 0.05, **p < 0.01, ***p < 0.001, determined by one-way ANOVA, Bonferroni's multiple comparisons test (A, B) and Tukey's multiple comparisons test (D, E).

Knockdown of uPAR in fibroblasts reduced the content of serpinE2 and collagen (Figure 3A). Knockdown of LRP1 also reduced the amount of serpinE2 and collagen in the cardiac fibroblasts (Figure 3B). Immunofluorescence assay showed that knockdown of uPAR or LRP1 reduced endocytosis of serpinE2 into cardiac fibroblasts (Figure 3C), respectively. Besides, ELISA assay also showed that knockdown of uPAR or LRP1 in cardiac fibroblasts significantly reduced levels of serpinE2 (Figure 3D). The similar trend of collagen secretion in the supernatant was also confirmed (Figure 3E). These results showed that the corresponding receptors on the cell membrane, LRP1 and uPAR, were involved in the endocytosis of serpinE2 into cardiac fibroblasts. The interaction between serpinE2 and uPAR, serpinE2 and LRP1 was then assessed by a Co-IP assay. uPAR, and LRP1 was observed in the anti- serpinE2 pull-down complex (Figure 3F). The results of protein Co-IP experiments showed that serpinE2 could not only bind to uPAR, but also serpinE2 could bind to LRP1. Taken together, endocytosis of serpinE2 mediated by LRP1 and uPAR is an important step for its regulation of collagen secretion in cardiac fibroblasts.

### SerpinE2 activates the ERK1/2 signaling pathway in cardiac fibroblasts

**Figure 4:**
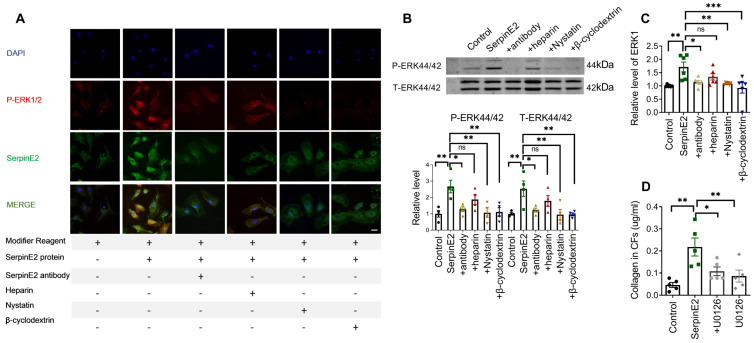
** SerpinE2 activates the ERK1/2 signaling pathway in cardiac fibroblasts. A.** Co-staining of p-ERK1/2 and serpinE2 in cardiac fibroblasts (*n* = 4, Scale bars = 20 µm). **B.** Relative expression level of phosphate-ERK1/2 in cardiac fibroblasts after administration of serpinE2 antibody, heparin, nystatin or β-cyclodextrin (*n*=4); **C.** Expression level of ERK1 was detected by ELISA assay after administration of serpinE2 antibody, heparin, nystatin or β-cyclodextrin (*n* = 6); **D.** Collagen expression level in CFs after treated with U0126 (*n* = 5). Data are presented as mean ± SEM. *p < 0.05, **p < 0.01, ***p < 0.001, determined one-way ANOVA, Bonferroni's multiple comparisons test (B-D).

It has been reported that serpinE2 activates the intracellular signal transduction pathway in mouse embryonic fibroblast (MEF) cells 14. Exogenous serpinE2 significantly increased p-ERK1/2 in cardiac fibroblasts, as detected by immunofluorescence. The p-ERK1/2 fluorescence intensity was weaker in the antibody-, nystatin-, and β-cyclodextrin-treated groups than that in the serpinE2 group (Figure 4A). Moreover, increased p-ERK1/2 protein levels in the serpinE2 group were also detected by western blotting. Compared with the serpinE2 group, the levels of p-ERK1/2 protein were significantly decreased in the antibody, nystatin and β-cyclodextrin applied groups (Figure 4B). Consistently, ELISA assays of ERK1 also verified the similar results (Figure 4C). Furthermore, to confirm whether ERK1/2 signaling pathway mediating the function of serpinE2, ERK1/2 inhibitor U0126 was applied to test if the level of collagen could be modulated in the presence of serpinE2. Obviously, the level of collagen was significantly decreased after addition of U0126 compared with serpinE2 group (Figure 4D), indicating that the endocytosis of serpinE2 could activate the ERK1/2 signaling pathway in cardiac fibroblasts.

### SerpinE2 activates β-catenin signaling pathway in cardiac fibroblasts

**Figure 5:**
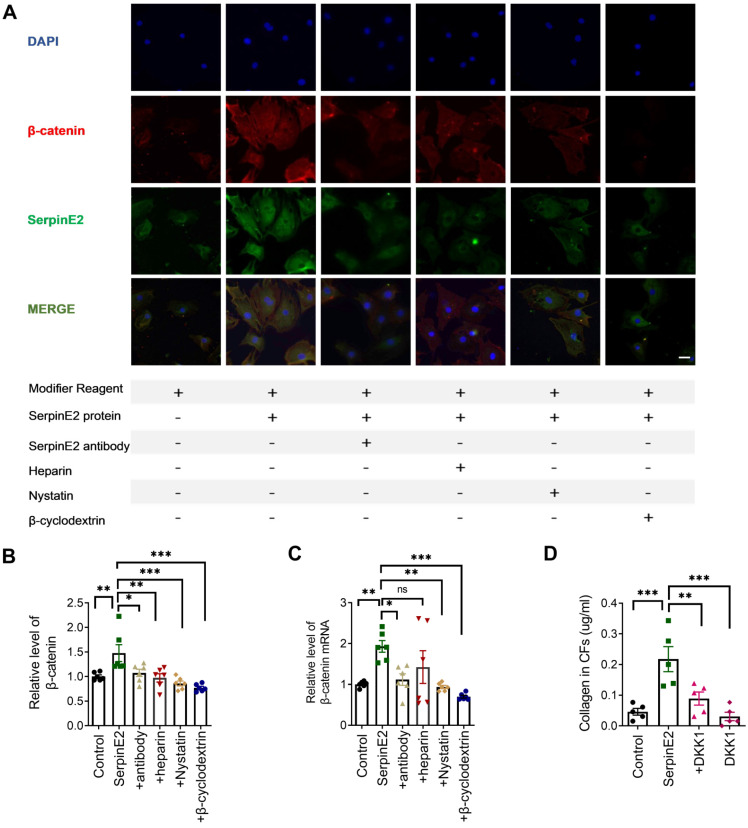
** SerpinE2 activates β-catenin signaling pathway in cardiac fibroblasts. A.** Co-staining of β-catenin and serpinE2 in cardiac fibroblasts (*n* = 4, Scale bars = 20 µm). **B.** Expression level of β-catenin was detected by ELISA assay after administration of serpinE2 antibody, heparin, nystatin, β-cyclodextrin (*n* = 6); **C.** mRNA level of β-catenin in cardiac fibroblasts after administration of serpinE2 antibody, heparin, nystatin, β-cyclodextrin (*n* = 6); **D.** Collagen expression level in CFs after treated with DKK1 (*n* = 5). Data are presented as mean ± SEM. *p < 0.05, **p < 0.01, ***p < 0.001, determined one-way ANOVA, Bonferroni's multiple comparisons test (B-D).

Immunofluorescence data showed that β-catenin expression was significantly increased in serpinE2 group, which was inhibited, respectively, in the antibody-, heparin-, nystatin- and β-cyclodextrin-treated groups (Figure 5A). ELISA results indicated that serpinE2 promoted the expression level of β-catenin, which was reversed by inhibition of serpinE2 endocytosis (Figure 5B). RT-PCR results showed that the mRNA levels of β-catenin were also increased in serpinE2 group and decreased in antibody-, nystatin-, and β-cyclodextrin-treated groups (Figure 5C). The results mentioned above have indicated that endocytosis of serpinE2 into cardiac fibroblasts may activate β-catenin signaling to promote the collagen secretion in cardiac fibroblasts. To confirm this, we administrated β-catenin inhibitor DKK1 to see if they could attenuate the increase in collagen level induced by serpinE2. Unsurprisingly, Inhibition of β-catenin signaling abolished the effect of serpinE2 on collagen production (Figure 5D). In sum, serpinE2 can be endocytosed into cardiac fibroblasts and then activated the downstream signaling pathway ERK1/2 and β-catenin to promote collagen secretion.

### Knockdown of serpinE2 attenuated cardiac fibrosis in TAC mouse model

**Figure 6:**
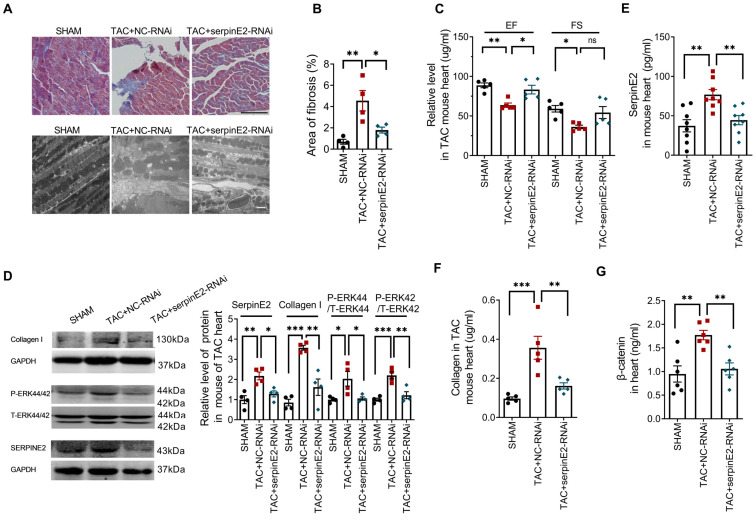
** Knockdown of serpinE2 attenuated cardiac fibrosis induced by TAC. A.** Masson staining showed the area of fibrosis (Scale bars = 100μm) and transmission electron microscope analysis of heart sections ( Scale bars = 2 µm); **B.** Fibrotic score was quantified by histological score from Masson staining (*n* = 4); **C.** Quantitative analysis of ejection fraction (EF) and fractional shortening (FS) of the heart (*n* = 5); **D.** Protein expression level of serpinE2, collagen, p-ERK44/42 and t-ERK44/42 (*n* = 4); **E-G.** Relative expression level of serpinE2, collagen and β-catenin detected by Elisa assay (*n* = 5-8); Data are presented as mean ± SEM. *p < 0.05, **p < 0.01, ***p < 0.001, determined one-way ANOVA, Bonferroni's multiple comparisons test (B-G).

To study the effect of serpinE2-mediated inhibition on cardiac fibrosis *in vivo*, we established TAC-induced mouse cardiac fibrosis model. Masson staining results showed that collagen deposition in myocardium was significantly increased in the tissues of TAC+NC-RNAi mouse heart compared with sham group, but was decreased in TAC+SerpinE2-RNAi group compared with TAC+NC-RNAi group; meanwhile, the results collected from transmission electron microscopy showed that, in TAC+NC-RNAi group, the shape of the myofilament was ambiguous and mitochondria were disordered while, these effects were ameliorated by knockdown of serpinE2 (Figure 6A-B). Besides, Echocardiography revealed that ejection fraction (EF) of the heart was markedly preserved in the serpinE2 knockdown group compared with the NC group after TAC (Figure 6C).

Western blotting results showed inhibited expression of serpinE2 along with the suppression of the level of p-ERK1/2 and collagen I in TAC+SerpinE2-RNAi group compared with those in the TAC+NC-RNAi group (Figure 6D). ELISA assays showed that TAC procedures could significantly up-regulated the expression level of serpinE2 in cardiac tissues. Knockdown of serpinE2 could significantly reduce the expression level of serpinE2 after TAC (Figure 6E). Meanwhile, knockdown of serpinE2 significantly lowered the expression level of collagen and β-catenin after TAC (Figure 6F-G). These results suggest that, inhibition of serpinE2 attenuates cardiac fibrosis after TAC and ERK1/2 and β-catenin signaling pathways are involved in this process.

## Discussion

**Figure 7:**
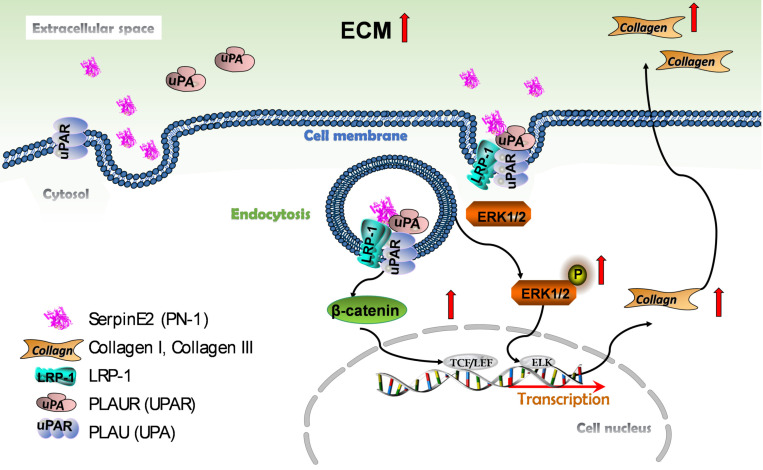
** Schematic model describing the effect and molecular mechanism of serpinE2 regulates collagen secretion in cardiac fibrosis.** SerpinE2 was endocytosed by cardiac fibroblasts through the membrane protein LRP and uPAR through lipid-raft dependent endocytosis, this process then activated the intracellular signaling ERK1/2 and β-catenin signaling pathway to promote synthesis of collagen in cardiac fibroblasts.

The current observations have demonstrated for the first time that serpinE2 can be internalized into cardiac fibroblasts due at least in part to the endocytosis process through directly interacted with the membrane protein LRP1 and uPAR. This process activates the ERK1/2 and β-catenin signaling pathways, and subsequently promotes collagen production in fibroblasts and induces cardiac fibrosis (Figure 7).

It has been demonstrated that the overexpression of serpinE2 promotes fibronectin synthesis in normal lung fibroblasts, and serpinE2 induces idiopathic pulmonary fibrosis (IPF) by directly regulating the expression of extracellular matrix proteins 15, 16. These are clear evidences showing the involvement of serpinE2 in the lung fibrosis, but whether it mediates cardiac fibrosis is unclear. Our previous study has demonstrated that serpinE2 is highly up-regulated in fibrotic cardiac tissues and knockdown of serpinE2 reduces TAC induced cardiac fibrosis level 6, 17. However, whether up-regulation of serpinE2 in cardiac fibroblasts contributes to cardiac fibrosis? If so, is this done by promoting collagen synthesis? In order to have a more comprehensive understanding of the function of serpinE2 in the heart, it is critical to answer these two questions.

In our current study, the internalization of serpinE2 was supported by the following two parts of data. The first, Dylight® experiment proved that exogenous serpinE2 protein can be translocated into the cardiac fibroblasts from extracellular space. Dylight®488 is a green fluorescent dye and it covalently binds to protein lysine or N-amino-terminal primary amine to label protein molecules, and does not affect the immune activity of proteins and antibodies, so it is often used to label proteins and antibodies 18. SerpinE2 is rich in lysine and arginine, containing a large amount of primary amines. Based on this principle, Dylight®488 green fluorescent dye is used to label exogenous serpinE2 protein. A quencher is added before immunofluorescence detection to quench the free Dylight®488 dye. Therefore, the green fluorescence can only be observed from serpinE2 labeled with Dylight®488. The second, the antibody against serpinE2 or heparin sodium were used in our study to block serpinE2 from binding to the membrane receptor. The official full name of serpinE2 is serine (or cysteine) peptidase inhibitor, clade E, member 2 (PN-1). SerpinE2 is a kind of secreted protein (397 amino acids) in extracellular space and poorly diffusible across membrane. Current evidence suggests that antibodies against serpinE2 can affect the function of serpinE2. The monoclonal antibodies binding specifically and with high affinity to human serpinE2/PN-1 abolish the inhibitory activity of protease on serpinE2/PN-1 19. The administration of serpinE2 antibody against serpinE2/PN-1 inhibits the upregulation of serpinE2 and effectively ameliorates airway remodeling in OVA-challenged mice 20. We speculate that serpinE2 may bind to certain membrane receptors (such as Upar or LRP) and be translocated into cardiac fibroblasts 8, 21. The polyclonal antibody against serpinE2 can capture some of its epitopes, which may affect its binding to membrane receptors. Studies have reported that soluble heparin was found to be a potent inhibitor of the binding of Th-PN-1/serpinE2 to the cell surface 22. The fact that heparin and free serpinE2 protein can easily form a complex strongly supports the involvement of cell surface heparins in the binding and internalization of uPA-serpinE2 23. Our results showed that both the antibody and heparin sodium could inhibit the internalization of serpinE2 and consequently affect its effect on collagen.

The membrane receptor involved in serpinE2 endocytosis in the heart remains unclear. LRP is involved in endocytosis 24 and recent progress has revealed that LRP is responsible for the endocytosis of PN1-protease complex in cerebellar 8. Besides, in human U937 monocyte cell line, PN-1 could form the PN-1-uPA complex which binds to uPAR on the cell membrane with the assistance of the 2-macroglobulin receptor and LRP1, then subsequently internalized in the cells 25. In our study, we want to explore if serpinE2 undergo endocytosis through LRP1 pathway into cardiac fibroblasts. Endocytosis can be divided into two main pathways - the classic, clathrin-mediated endocytic pathway and the non-classic, clathrin-independent, but lipid-raft dependent route. Clathrin-independent (lipid-raft dependent) internalization routes are sensitive to cholesterol depletion 26. LRP1 is known to be an endocytic receptor 27, which related to the lipid-raft dependent route 28. So, cholesterol-depleting agents (β-cyclodextrin and Nystatin) were used to inhibit the lipid-raft dependent endocytosis 29, 30. Furthermore, since β-cyclodextrin and Nystatin inhibit lipid-raft dependent endocytosis by depleting cholesterol on the cell membrane, the content of cholesterol is crucial for detecting the endocytosis of serpinE2. Reduction of serpinE2 in CFs after treatment with β-cyclodextrin or Nystatin may not necessarily indicate the endocytosis of serpinE2 since β-cyclodextrin or Nystatin has many non-specific effects and there was no direct evidence that cholesterol was involved. We need to detect when there is sufficient cholesterol around the cells to exclude the influence of cholesterol. Our experiment demonstrated that exogenously administered serpinE2 was internalized into cardiac fibroblasts, β-cyclodextrin or Nystatin inhibit the internalization of serpinE2 under both normal condition and cholesterol condition. Eventually, knockdown of LRP1 or uPAR also reduce the endocytosis of exogenous serpinE2 into cardiac fibroblasts and decrease expression level of collagen and serpinE2 can directly bind to LRP1 and uPAR. Similar to previous studies, in current observations, layer by layer proof was adopted to confirm serpinE2 can directly bind to LRP1 and uPAR which can mediate the endocytosis of serpinE2 in cardiac fibroblasts.

In breast cancer cell lines, serpinE2 can bind to LRP1 on the cell membrane and activate the ERK1/2 signaling pathway 31. Besides, serpinE2 can also activate ERK1/2 signaling in mouse embryonic fibroblasts, intestinal epithelial cells, and human chondrocytes 14, 32. Our current study is consistent with the above results, that serpinE2 could be translocated into cardiac fibroblasts through endocytosis and then activate the ERK1/2 signaling pathway to promote collagen production in the heart. In our previous study it was found that the MEK1/2 - ERK1/2 signaling promoted the expression of serpinE2 via transcription factors ELK1 in myocardial fibroblast 6. Therefore, we get the conclusion that serpinE2 is regulated by ERK1/2 and can also regulate the expression of ERK1/2, which forming a positive feedback regulation. The results above have fully demonstrated that exogenous serpinE2 could be translocated into cardiac fibroblasts through endocytosis and then activate the Erk1/2 and β-catenin signaling pathway to promote collagen production. Wnt signaling pathway significantly participates in cardiac fibrosis and CFs activation 33. The canonical Wnt signaling pathway is mediated by interaction of β-catenin with the T-cell factor/lymphoid enhancer factor (TCF/LEF) transcription factors and subsequent transcription activation of Wnt-target genes.

Collectively, this study provides novel evidence to strength our understanding that exogenous serpinE2 once to be endocytosed into cardiac fibroblasts with LRP1 and uPAR assistance could activate the ERK1/2 and β-catenin signaling pathway, which promotes fibroblast collagen production/deposition and induces cardiac fibrosis. Thus, targeting endocytosis of serpinE2 may shed new light for clinical management of cardiac fibrosis.

## Figures and Tables

**Table 1:** Primers used for the qRT-PCR analysis

RNA name	Primers from 5' to 3'
Collagen I-F	AAGAAGACATCCCTGAAGTCA
Collagen I-R	TTGTGGCAGATACAGATCAAG
Collagen III-F	TTGGGATGCAGCCACCTTG
Collagen III-R	CGCAAAGGACAGATCCTGAG
β-catenin-F	ATCATTCTGGCCAGTGGTGG
β-catenin-R	GACAGCACCTTCAGCACTCT
α-SMA-F	GTACCACCATGTACCCAGGC
α-SMA-R	GCTGGAAGGTAGACAGCGAA
GAPDH-F	GGGGCTCTCTGCTCCTCCCTG
GAPDH-R	CGGCCAAATCCGTTCACACCG
